# Genetic characterization of the oxytocin-neurophysin I gene (*OXT*) and its regulatory regions analysis in domestic Old and New World camelids

**DOI:** 10.1371/journal.pone.0195407

**Published:** 2018-04-02

**Authors:** Alfredo Pauciullo, Danlami Moses Ogah, Marco Iannaccone, Georg Erhardt, Liliana Di Stasio, Gianfranco Cosenza

**Affiliations:** 1 Department of Agricultural, Forest and Food Science, University of Torino, Grugliasco (TO), Italy; 2 Department of Animal Science, Nasarawa State University, Keffi, Shabu-Lafia, Nigeria; 3 Department of Agricultural Sciences, University of Naples Federico II, Portici (NA), Italy; 4 Department of Animal Breeding and Genetics, Justus-Liebig-University Giessen, Giessen, Germany; Institute of Farm Animal Genetics, GERMANY

## Abstract

Oxytocin is a neurohypophysial peptide linked to a wide range of biological functions, including milk ejection, temperament and reproduction. Aims of the present study were a) the characterization of the *OXT* (Oxytocin-neurophysin I) gene and its regulatory regions in Old and New world camelids; b) the investigation of the genetic diversity and the discovery of markers potentially affecting the gene regulation. On average, the gene extends over 814 bp, ranging between 825 bp in dromedary, 811 bp in Bactrian and 810 bp in llama and alpaca. Such difference in size is due to a duplication event of 21 bp in dromedary. The main regulatory elements, including the composite hormone response elements (CHREs), were identified in the promoter, whereas the presence of mature microRNAs binding sequences in the 3’UTR improves the knowledge on the factors putatively involved in the *OXT* gene regulation, although their specific biological effect needs to be still elucidated. The sequencing of genomic DNA allowed the identification of 17 intraspecific polymorphisms and 69 nucleotide differences among the four species. One of these (MF464535:g.622C>G) is responsible, in alpaca, for the loss of a consensus sequence for the transcription factor SP1. Furthermore, the same SNP falls within a CpG island and it creates a new methylation site, thus opening future possibilities of investigation to verify the influence of the novel allelic variant in the *OXT* gene regulation. A PCR-RFLP method was setup for the genotyping and the frequency of the allele C was 0.93 in a population of 71 alpacas. The obtained data clarify the structure of *OXT* gene in domestic camelids and add knowledge to the genetic variability of a genomic region, which has received little investigation so far. These findings open the opportunity for new investigations, including association studies with productive and reproductive traits.

## Introduction

Nowadays, six species of camelids exist in the world which include both domestic (*Camelus dromedarius*, *Camelus bactrianus*, *Lama glama*, *Vicugna pacos*) and wild animals (*Camelus ferus*, *Lama guanicoe*, *Vicugna vicugna*). Their origin traces back to the Eocene (40–45 mya) when the first ancestors of the Camelidae family were found in North America [1].

Approximately 37 million camelids are kept worldwide [2]. The majority (about 75%) are Old World camelids (dromedary and bactrian) distributed in the Afro-Asian dryland, the former mainly in Somalia and the latter in Mongolia/China. Conversely, the Andean highlands are the natural habitat of New World camelids (llama, alpaca, guanaco and vicuna) mainly distributed in Peru, Chile and Argentina.

These species represent an important economic source for the rural populations of those areas, having a fundamental socio-cultural role in the development of pastoral zones. Camels are mainly kept for transport and milk production. As dairy animals, they often provide the staple food in pastoralist societies [3,4]. Conversely, there is no historic tradition of milking llamas and alpacas, which have not been exploited as source of this product. In fact, South American camelids (SACs) are kept as multipurpose animals, mainly for fleece, meat and transport, and often they are used as trekking animals, contributing to the development of the agro-touristic business [5–7].

Traits like milk yield, temperament and reproduction are of fundamental interest for the improvement of camelids breeding in many arid and semiarid areas, and their regulation may also be associated with the oxytocin release. Oxytocin plays an important role in the regulation of various physiological functions. It is an indispensable hormone for milk ejection from the mammary gland [8], it promotes social bonds and influences temperament in adult animals [9,10], it stimulates uterine smooth muscle contraction during labour/parturition [11]. Moreover, the oxytocin has other roles, including the control of oestrous cycle length, the follicle luteinization and the ovarian steroidogenesis. Furthermore, it acts as a neurotransmitter in the central nervous system (CNS) and plays a role in different processes like cognition, tolerance, adaptation, complex sexual and maternal behavior, as well as in the regulation of water excretion and cardiovascular functions [12].

The oxytocin-neurophysin I gene (*OXT*) has been sequenced and fully characterized in many species, including domestic ruminants [13–15], and polymorphisms have been associated with milk yield and flow [16], which are of great interest also in camelids. In fact, camel milk has been characterized with regard to its protein fractions [17–20] and biological active peptides [21], moreover a detailed description of llama milk proteins has been recently provided [22] and the genetic basis of casein synthesis has been elucidated [23,24].

Despite the considerable interest for the *OXT* gene and its function for the milk ejection, no information at DNA and protein level has been reported so far in camelids and no studies have been carried out to investigate genetic polymorphisms. Although the genome sequencing has been completed for the dromedary and bactrian camels, as well as for the alpaca [1,25], the annotation is still incomplete. Furthermore, the homology of orthologous gene sequences with camelids genomes showed gaps in that DNA region.

Based on all these considerations, an investigation was undertaken to explore the genetic diversity at the *OXT* in the four domestic camelids. We provide for the first time the full characterization and annotation of the gene regulatory regions and report on first polymorphisms, which could affect the *OXT* gene regulation.

## Materials and methods

### Ethics statements

No animals were used in the present study. The samples used herein belonged to DNA collections available from past studies [19,23,24,26] already approved by different ethic committees. Therefore, according to the Committee on the Ethics of Animal Experiments of the University of Torino (D.R. n. 2128 released on 06/11/2015) further ethics approval was not required.

### DNA samples

Samples used in this study belong to DNA collections of research groups in different areas of the world. In particular, dromedary DNA were provided by Nasarawa State University (Nigeria), bactrian camels DNA came from Justus-Liebig-University (Germany), whereas alpacas and llamas DNA belong to the collections of University of Turin (Northern Italy) and Justus-Liebig-University (Germany).

The original biological tissue used for DNA isolation was blood. Individual samples from unrelated animals belonging to different farms were collected during routine treatments according to national rules on animal welfare of the country of origin. Details on DNA isolation procedure were reported in previous studies [19,23,24,26]. Briefly, a blood drop for each dromedary was spotted on individual cellulose filter paper, air dried at room temperature, transported to the laboratory and then used for DNA isolation. Conversely, fresh blood samples were collected from the other camelids.

The filter paper was soaked (56°C, overnight) in 500 μl sodium-Tris-EDTA buffer with 10 μl proteinase K (10 mg/ml) in presence of sodium dodecyl sulfate (SDS). DNA was isolated from the emerging lysis according to the procedure described by [27] and resuspended in 100 μl TE buffer pH 7.6 (10 mM Tris, 1mM EDTA). Conversely, the fresh samples were treated according to the Spin Blood Mini Kit (Invitek, Germany).

Concentration and OD_260/280_ ratio of the DNA samples were measured with the Nanodrop ND-1000 Spectrophotometer (Thermo Fisher Scientific Inc., Waltham, MA, USA). Average concentrations were 50 ng/μl whereas a ratio higher than 1.8 was recorded for all the DNA samples.

Twelve DNA samples (three samples from each species) were chosen for sequencing the whole *OXT* gene. In addition, 20 dromedaries, 68 alpacas and 20 llamas’ DNA samples were used for genetic polymorphism discovery and genotyping.

### PCR amplification and sequencing

All primers for the amplification and sequencing (Table 1) were designed by DNAsis-Pro software (Hitachi Software Engineering Co., San Bruno, CA, USA) using as template the Contig_15904_16 belonging to an unannotated genome fragment of dromedary (Genbank ID: LSZX01119753) and putatively containing the target gene based on the gene homology with the *OXT* gene sequences of domestic ruminant (GenBank IDs: X00502; AM234538; LT592265; LT592266).

**Table 1:** Primer sequences, annealing temperature (T_a_) and amplicon size used for the sequencing and genetic diversity discovery at *OXT* locus in domestic camelids.

Region amplified	Primer	Sequence	T_a_ °C	Amplicon size
5’ Flanking Region	OXT 5end F	5’-AGCACCTCCCTGTTCAT-3’	65,3°C	957 bp
	OXT prom R	5’-TGTCTAAGAGGGCCGC-3’		
Promoter–Exon 3	OXT prom F	5’-CGGCACTCGCTATCATC-3’	65,0°C	976 bp
	OXT Ex 3 R	5’-TCAGTGCTGGGAGAAGG		
3’ Flanking Region	OXT Ex 3 F	5’-CGCCGAGCCCGCCTG-3’	60,0°C	463 bp
	OXT 3end R	5’-AGCCTTCTTCCCAATGTC-3’		

All the primers were designed using the contig_15904_16 as template (Genbank ID: LSZX01119753).

PCR mixtures were prepared according to [23], whereas the thermal conditions applied for the amplification were: 97°C for 4 min, 35 cycles at 97°C for 45 s, annealing for 45 s with temperatures depending on the amplicon (Table 1), and extension at 72°C for 45 s. A final extension was carried out at 72°C for 5 min.

The amplified products were analysed by electrophoresis on agarose gel in 0.5X tris boric acid EDTA buffer (TBE), purified using NucleoSpin^®^ PCR Clean-up (Macherey-Nagel GmbH, Düren, Germany) and sequenced in both directions at Microsynth GmbH (Vienna, Austria) by classic Sanger technology.

SNP discovery in dromedary, alpacas and llamas was accomplished by the sequencing of additional 60 DNA samples in total (20 for each species) for the whole *OXT* gene including the promoter and the 3’ flanking region.

### Genotyping of the polymorphism g.622C>G in the alpaca promoter

A PCR-RFLP (restriction fragment length polymorphism) method was developed for the SNP MF464535:g.622C>G found in the promoter region of the alpaca *OXT*. A DNA fragment 957 bp long was amplified using the primers described in Table 1 for the amplicon 1.

Five μl of each amplicon was digested with 1U of FastDigest *Bfo* I endonuclease (5’-RGCGC↓Y-3’) (Thermo Scientific) for 15 min at 37°C. The digested products were analysed by electrophoresis in 1.5% agarose gel in 0.5X TBE buffer and stained with Sybr Green (Sigma-Aldrich).

### Bioinformatic and statistical analysis

Homology searches, comparison among sequences, and multiple alignments were performed by DNAsis-Pro (Hitachi Software Engineering Co., San Bruno, CA, USA). Prediction signals and combined cleavage sites for leader peptides was determined by SignalP V4.1 software (www.cbs.dtu.dk/services/SignalP). Splice site prediction was performed by NNSPLICE ver. 0.9 (http://www.fruitfly.org/seq_tools/splice.html), whereas branch point prediction was carried out by SVM-BP finder software (http://regulatorygenomics.upf.edu/Software/SVM_BP/). The putative transcription factor binding sites at the promoter were searched by Transfact 7.0 software considering the most stringent condition of analysis (85% as minimum binding score and 100% similarity of the sequence to consensus matrix), whereas the 3’-flanking region was analysed for potential microRNA sequences by using the TargetScan method [28]. Allelic frequencies and Hardy-Weinberg equilibrium were evaluated for the SNP MF464535:g.622C>G using PopGene software ver. 1.32 (University of Alberta, Canada).

## Results and discussion

### *OXT* gene structure in domestic camelids

Whole genome assemblies on scaffold-level are available for dromedary [1,25], Bactrian camel [29], and alpaca [30], in addition to other camelid genome projects in databases. Often the low coverage assembly and the tentative annotations built on the human genome led to redundant information, exon losses and errors in gene annotations. This is even more evident for less investigated species like camelids. This is well exemplified by the β-casein in the alpaca genome. In fact, the exon 3 is out-spliced in human and, since the human genome is used as reference, this exon has not been annotated in alpaca. However, the DNA sequence encoding for the exon 3 can be easily retrieved approximately 130 bp upstream of the provided *Vicugna pacos* genomic sequence [23]. This example highlights the need to gain more experimental data to help the annotation of the new investigated species.

**Fig 1:**
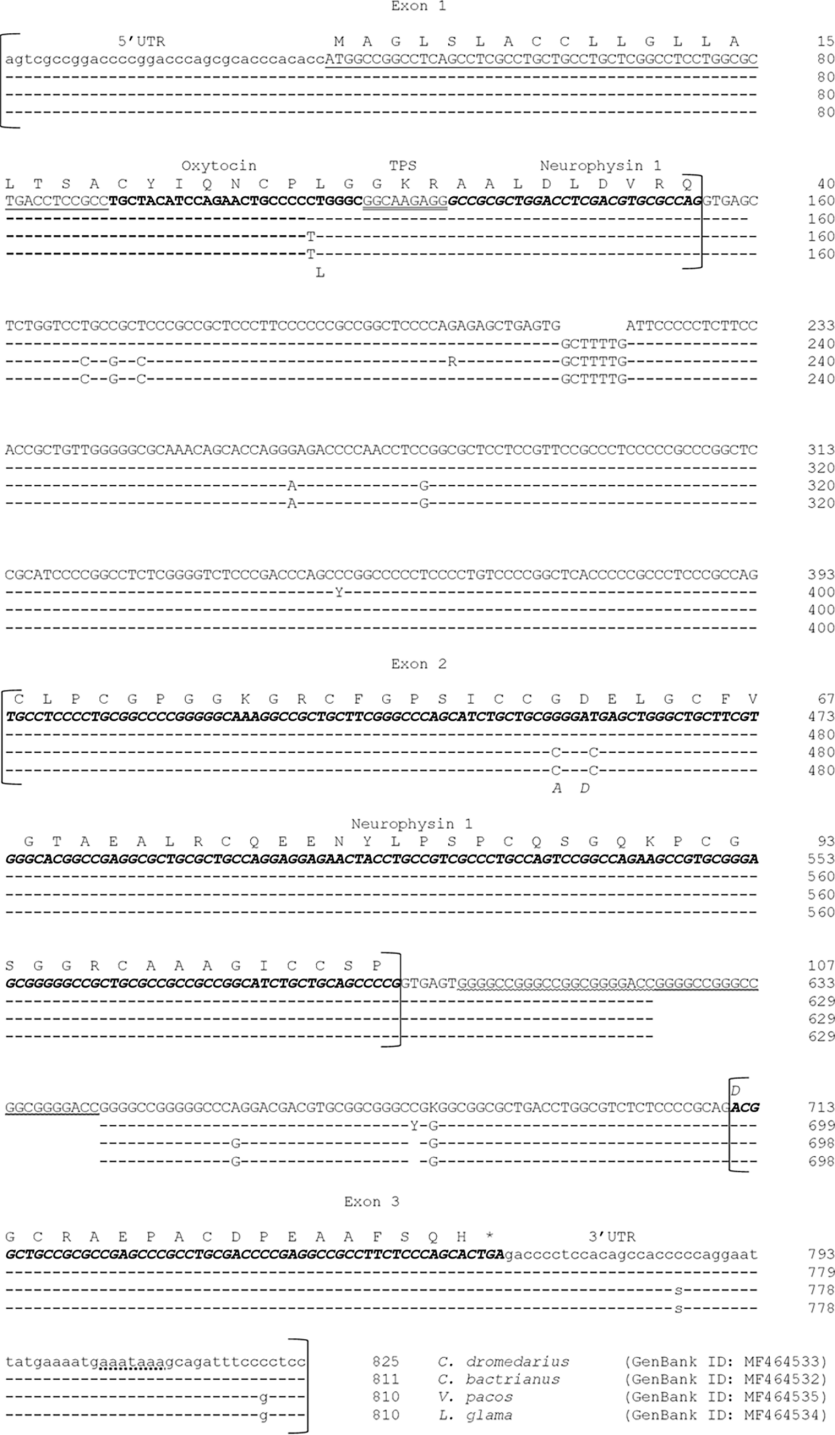
*OXT* gene in domestic camelids. Comparative alignment of the complete nucleotide (nt) sequences of oxytocin-neurophysin I encoding (*OXT*) gene in domestic camelids. Numbering is relative to the first nucleotide of the first exon (+1) and dashes represent nt identical to those in the first line. In lower cases the 5’- and 3’- Un-Translated Regions (UTR), the polyadenylation signal is dot-underlined. The coding region corresponding to the signal peptide is underlined, whereas the sequence coding for the nonapeptide hormone is indicated in bold, and the neurophysin I is in bold italics. The tripeptide processing signal (GKR) is double underlined and asterisks indicate the stop codon. The duplication event of 21bp in *C*. *dromedarius* is wave-underlined. Polymorphic sites within the investigated samples are indicated with R = A/G, S = C/G and Y = C/T.

In this context, we sequenced the whole gene encoding the oxytocin-neurophysin I (*OXT*) plus 965 nucleotides at the 5’flanking region and 295 bp at the 3’ flanking region in the four domestic camelids (*C*. *dromedarius*, GenBank ID: MF464533; *C*. *bactrianus*, GenBank ID: MF464532; *L*. *glama*, GenBank ID: MF464534 and *V*. *pacos*, GenBank ID: MF464535). On average, the gene extends over 814 bp, ranging between 825 bp in dromedary, 811 bp in bactrian camel and 810 bp in llama and alpaca (Fig 1) and it includes 471 bp of exonic regions and 343 bp (on average) of intronic regions. The gene contains only 3 exons, respectively 154 bp (exon 1), 202 bp (exon 2) and 115 bp (exon 3), and 2 introns of 239/246 bp (intron 1) and 93/115 bp (intron 2). The 5’ untranslated region (UTR) includes the first 34 nucleotides of the first exon, while the 3’UTR includes the last 59 nucleotides of the exon 3.

The ORF (Open Reading Frame) region is 375 bp long and it codes for the signal peptide (19 amino acids—nucleotides 34–90) and for the 106 amino acids of the mature oxytocin-neurophysin I complex. In particular, the first 9 amino acids of the complex (from nucleotide 91 to 117 of the exon 1) belong to the nonapeptide hormone, followed by the tripeptide processing signal (GKR) (from nucleotide 118 to 126), and finally 94 amino acids of the neurophysin I (last 27 bp of the exon 1, complete exon 2 and first 54 bp of the exon 3).

The translation stop codon TGA is located between the nucleotides 54–56 of the exon 3, whereas the polyadenylation signal (aaataaa) is located between the nucleotides 94–100 of the same exon. Splice donor and acceptor consensus sequences conforming to the GT/AG rule were identified at the exon/intron boundaries.

Interestingly, the duplication of 21 bp (MF464533:g.1567_1608dupGGGGCCGGGCCGGCGGGGACC) was observed only in the dromedary *OXT* sequence between the nucleotides 1567–1608 of the intron 2 (Fig 1). This event is unique also in comparison with the domestic ruminants. Duplication events are not rare for the *OXT* gene, being itself the result of an ancestral gene duplication also involving the vasopressin gene [12]. Examples of repeated DNA sequences were recently reported in sheep *OXT* intron 1 and in goat 3’ flanking region [13]. The duplication observed in dromedary was monomorphic, therefore its fixation is responsible of the sequence expansion of the intron 2 (115 bp long) compared with that of the other camelids (94 bp in bactrian camel and 93 bp in llamas and alpacas). Conversely, the dromedary showed a shorter intron 1 for the deletion of an epta-nucleotide (MF464533:g.1184_1191delGCTTTTG) (Fig 1).

### Similarities and differences of the *OXT* gene in camelids and ruminants

Overall, the *OXT* gene in domestic camelids shares a similar organization with the ruminant counterpart [13,14,31], although showing some differences both at exonic and intronic levels. The cDNA is 41 bp shorter than the bovine one (471 bp *vs* 512 bp). The main differences were detected in the exon 3 corresponding to the 3’ untranslated region (S1 Fig). A similar situation has been already described in genes controlling milk proteins in camelids. For instance, compared to cattle, the 3’ UTR of the *CSN2* is shorter in camel (317 bp *vs* 323 bp) [19] and the *CSN1S2* is shorter in llamas (981 bp *vs* 1024 bp) [23].

The intron sizes for the *OXT* gene are rather different between domestic camelids and domestic ruminants. The intron 1 is shorter for the first group (239/246 bp) compared to the bovine counterpart (309 bp), whereas the situation is opposite for the intron 2, which is slightly longer (93 to 115 bp) in camelids than in cattle (90bp). These differences result in lower exon/intron size ratios for the domestic camels (from 1:1.33 in dromedary to 1:1.38 in the other investigated camelids) than that observed in cattle (1:1.28). In total, the level of similarity found with *OXT* gene of the domestic ruminant was about 70.0%.

### SNP discovery and analysis of genetic diversity

SNP discovery was accomplished by re-sequencing the whole gene, including the flanking regions, for 60 animals belonging to dromedary, alpacas and llamas (20 samples each). The intra-species comparison of the sequences showed a total of 17 polymorphic sites (9 transitions, 7 transversions and one deletion). The New world camelids were more polymorphic (14 SNP) than the Old world species (3 SNP); the alpaca *OXT* was the most variable with 8 SNP found (Table 2). The lower genetic variability found in Old World compared to New World camelids confirms the reduction of genetic diversity in the former species as consequence of at least two severe bottlenecks which negatively affected the effective population size [32].

**Table 2:** Genetic diversity detected by the sequencing of the *OXT* gene and its regulatory regions in domestic camelids (*C*. *dromedarius*, *C*. *bactrianus*, *V*. *pacos*, *L*. *glama*).

Species	GenBank ID	Location	Position	Nucleotide
*C*. *dromedarius*	MF464533	Intron 2	1644	K
*C*. *bactrianus*	MF464532	Intron 1	1321	Y
		Intron 2	1628	Y
*V*. *pacos*	MF464535	Promoter	39	W
			326	Y
			465	R
			622	S
			668	R
		Intron 1	1120	R
		Exon 3 (3’UTR)	1682	S
		3’flanking region	1849	S
*L*. *glama*	MF464534	Promoter	604	R
			717	R
		Exon 3 (3’UTR)	1731	S
		3’flanking region	1898	S
			1948–1950	del G
			2001	R

Note: Y = C/T; R = A/G; S = C/G, K = G/T, W = A/T

Numbering is relative to the GenBank accession numbers specific for each species.

Most of the polymorphisms were found in the regulatory regions, 7 in the promoters and 4 in the 3’ end (Table 2), while no genetic variability was detected in the coding regions. At intron level four SNP were found, but none of them affected key sites of the spliceosome machinery (acceptor sites, branch points, polypyrimidine tracts, donor sites). Therefore, these mutations are not expected to influence the gene expression.

The comparison among the *OXT* sequences of the four species showed 69 additional polymorphic sites (Table 3). Almost all the detected SNPs (85.5%) differentiate the Old from the New World camelids. Five nucleotide differences were found at the exon level. One of these, located in the exon 2, is a transversion g.1417C>G (taking as reference the dromedary sequence) which is responsible for the amino acid replacement p.Gly60Ala within the neurophysin coding region (residue 29 of the neurophysin). The glycine is typical of the Old World camelids, whereas the alanine characterises the SACs. Furthermore, two transitions are synonymous (g.1078C>T, p.Leu27 = at the exon 1; and g.1421T>C, p.Asp61 = at the exon 2) and two SNP fall within the 3’ untranslated region (Table 3).

**Table 3:** Polymorphisms detected by the comparison among the complete sequences of *OXT* gene and the regulatory regions of domestic camelids investigated in the present study (*C*. *dromedarius*, *C*. *bactrianus*, *V*. *pacos*, *L*. *glama*).

		*C*. *dromedarius*		*C*. *bactrianus*		*V*. *pacos*		*L*. *glama*	
		(MF464533)		(MF464532)		(MF464535)		(MF464534)	
		Position	Nucleotide	Position	Nucleotide	Position	Nucleotide	Position	Nucleotide
			(amino acid)		(amino acid)		(amino acid)		(amino acid)
Promoter		10	T	10	G		*	8	T
		25	T	25	T		*	23	C
		33	G	33	G		*	31	A
		65/67	AAA	65/67	AAA	15/17	GGG	63/65	GGG
		68	A	68	A	17/18	-	66	G
		71	A	71	A	20	C	69	C
		71/72	—	71/72	—	21/22	CC	70/71	CC
		88	T	88	T	39	W	88	T
		109	C	109	C	60	T	109	T
		135	C	135	C	86	T	135	T
		153	T	153	T	104	A	153	A
		168	T	168	T	119	C	168	C
		182	C	182	C	133	T	182	T
		228	C	228	C	178/179	-	227–228	-
		236	G	236	G	186	A	235	A
		262/264	ATG	262/264	ATG	211/212	—	260/261	—
		291	T	291	T	238	C	287	C
		311	G	311	G	258	A	307	A
		377	A	377	A	324	C	373	C
		379	C	379	C	326	Y	375	C
		388	G	388	G	335	A	384	A
		392	A	392	A	339	G	388	G
		443	T	443	T	390	C	439	C
		468	A	468	T	415	T	464	T
		473	G	473	G	420	T	469	T
		492	A	492	A	439	G	488	G
		509	A	509	A	456	G	505	G
		518	G	518	G	465	R	514	G
		540	G	540	G	487	A	536	A
		550	T	550	T	497	C	546	C
		566	A	566	A	513	C	562	C
		599	G	599	C	546	G	595	G
		608	A	608	A	555	A	604	R
		622	G	622	G	569	A	618	A
		624	A	624	A	571	C	620	C
		627	C	627	C	574	T	623	T
		630	T	630	T	577	C	626	C
		650	G	650	G	597	A	646	A
		675	C	675	C	622	S	671	C
		687	A	687	A	632	G	681	G
		716	T	716	T	663	A	712	A
		721	A	721	A	668	R	717	R
		722	C	722	C	669	T	718	T
		732	C	732	A	679	C	728	C
		740	G	740	G	687	A	736	A
		795	T	795	T	742	C	791	C
		819	C	819	C	766	G	815	G
		829	C	829	T	776	C	825	C
		841	T	841	T	788	C	837	C
		943	G	943	G	890	C	939	C
		953	G	953	G	900	C	949	C
		960	C	960	T	907	C	956	C
OXT	Exon 1	1078	C	1078	C	1025	T	1074	T
			p.27Leu		p.27Leu		p.27Leu		p.27Leu
	Intron 1	1134	T	1134	T	1081	C	1130	C
		1137	C	1137	C	1084	G	1133	G
		1140	T	1140	T	1087	C	1136	C
		1173	G	1173	G	1120	R	1169	G
		1229	G	1236	G	1183	A	1232	A
		1243	C	1250	C	1197	G	1246	G
		1314	C	1321	Y	1268	C	1317	C
	Exon 2	1417	G	1424	G	1371	C	1420	C
			*p*.*60Gly*		*p*.*60Gly*		*p*.*60Ala*		*p*.*60Ala*
		1421	T	1428	T	1375	C	1424	C
			p.61Asp		p.61Asp		p.61Asp		p.61Asp
	Intron 2	1623	A	1609	A	1556	G	1605	G
		1642	C	1628	Y	1574/1575	-	1623/1624	-
		1644	K	1630	G	1576	G	1625	G
	Exon 3 (3’UTR)	1750	C	1736	C	1682	S	1731	S
		1786	C	1772	C	1718	G	1767	G
3’ flanking region		1895	G	1895	G	1819/1820	-	1868/1869	-
		1907	T	1907	T	1831	C	1880	C
		1925	C	1925	C	1849	S	1898	S
		1987	C	1987	C	1911	T	1960	T
		1996	T	1996	G	1920	G	1969	G
		2011	G	2011	G	1935	A	1984	A
		2028	G	2028	G	1952	G	2001	R
		2036/2040	AGAGG	2036/2040	AGAGG	1960/1964	CTCCA	2009/2013	CTCCA
		2043/2044	CA	2043/2044	CA	1967/1968	GG	2016/2017	GG
		2046	T	2046	T	1970	C	2019	C
		2052/2054	GTC	2052/2054	GTC	1976/1978	CCA	2025/2027	CCA
		2057	A	2057	A	1981	G	2030	G
		2079	G	2079	G	2012	A	2061	A
			*	2086	G	2019	G	2067/2068	-

Numbering is relative to the GenBank accession numbers specific for each species. Mutations detected in the investigated samples (W = A/T, Y = C/T; R = A/G; S = C/G, K = G/T) are reported in bold. Grey cells identify nucleotides identical to the sequence of the *C*. *dromedarius OXT* taken as reference. Dashes indicate deleted nucleotides, asterisks show unavailable sequences.

It is also interesting to notice that alpacas and llamas are characterized by three common polymorphisms (Table 3), the first in the promoter (g.668A>G and g.717A>G), the second in the 3’UTR (g.1682C>G and g.1731C>G) and the last in the 3’ flanking region (g.1849C>G and 1898C>G). A similar event was observed also in the sheep and goat *OXT* for the transition LT592265:g.438T>C, where convergent evolution, genetic introgression or adaptive genetic variation in the form of Trans-Specific Polymorphism (TSP) have been considered as possible evolutionary mechanism responsible for this event [13]. However, unlike sheep and goat, where evolutionary divergence has slowly developed and finally fixed by a different arrangement and number of chromosomes (sheep 2n = 54 and goat 2n = 60), which *de facto* makes hybridization very difficult in nature [33], the hybridization between alpacas and llamas is much more extensive [5]. This is due to the conserved structure of their karyotypes, both made of 74 chromosomes, which makes also their offspring fertile and backcrossing to either parental species possible [34]. As a consequence, genetic introgression in SACs might be considered as the main mechanism responsible for the common polymorphic sites observed herein. This is consistent with the genetic introgression observed in Old world camelids [32] and with the data reported by Kadwell et al. in 2001 [5] on substantial mitochondrial introgression highlighted in the alpaca by cytochrome *b* sequencing and nuclear introgression detected in the llamas using microsatellite analysis.

### Analysis of the regulatory regions

The analysis of the regulatory regions (promoter and 3’-end) provides an important contribution for the evaluation of the factors involved in the regulation of gene expression. In fact, the promoter contains consensus sequences, which bind transcription factors enhancing the gene expression. The 3’-flanking region plays an important role in the repression of the gene expression mediated by microRNA [35]. Therefore, any polymorphism falling in these regions might affect binding sites and modify the transcription rate or the mRNA stability and, consequently, the amount of the protein. Therefore, we decided to extend the sequencing to the 5’-flanking region (more than 950 bp upstream exon 1) and to the 3’-flanking region (more than 300 bp downstream exon 3) for SNP discovery purposes and for the analysis of the putative regulatory regions.

#### Sharing of transcription factor SP1 between domestic camelids and ruminants

Homology level and similar locations of the main putative transcription binding sites were already reported for the *OXT* promoters of domestic ruminants [12–15]. Similar mechanisms of regulation are supposed for ruminants. Therefore, we compared the *OXT* promoter sequences of domestic camelids with the cattle (AB481096), buffalo (AM234538), sheep (LT592265) and goat (LT592266) homologous sequences to explore possible similarities and differences.

Bioinformatic analysis using Transfact 7.0 software found that the *OXT* promoter region in camelids contained at least 18 high-scoring (85%-100%) putative binding sites (S2 Fig). Half of them are represented by the transcription factors SP1, which regulates target genes by steroid hormone receptors [36]. The high number of these motifs is due to the considerable distribution of GC-rich sequences, which in several gene promoters are *cis*-acting elements for an increasing number of ligand-activated receptors, that interact with SP1 and related proteins [36,37]. It is interesting to notice that only one SP1 (-122/-113) is shared between domestic camelids and ruminants. The other 8 SP1 are only partially shared among the camelids and in total the SACs showed one more SP1 than Old World camelids.

#### Interaction between the *OXT* gene and hormone receptors

Several members of the nuclear receptor family, including many orphan receptors, could interact with the *OXT* gene and regulate its expression. For instance, hexanucleotide AGGTCA motifs and its variations (direct or inverted repeats) have been reported as part of binding sites of estrogen receptors (ERα and ERβ), thyroid hormone receptor (THRα) and retinoic acid receptors RARα and RARβ [38,39]. In this study, two putative estrogen response elements (ERE) half sites were identified in position -85/-81 and -105/-101, but similarly to human, the authentic palindromic ERE (GGTCA—TGACC) was not found. This result suggests that the oestrogen effect on *OXT* transcription could be indirect rather than direct, as it was already observed in *OXTR* [40]. In fact, such ERE half sites fall within a minor composite hormone response element (CHRE) as reported in rat [41] and they can act synergistically with the full CHRE [12] found upstream in position -165/-153.

The CHRE is considered as the most conserved element essential for the regulation of *OXT* gene expression. In fact, the deletion of this region in co-transfection experiments resulted in complete loss of most of the responsiveness to estrogen and retinoid acid [42]. Differently from ruminants, the consensus sequence of the CHRE in the domestic camelids (GGTGACCTTGACC) corresponds perfectly to those of humans and rats. Therefore, a regulation through the same activators, like estrogen receptor-*β*, thyroid hormone, retinoic acid, steroidogenic factor I [43], and repressor, like chicken ovoalbun upstream promoter transcription factor I (COUP-TFI) [44], might also be working in camelids.

#### Additional consensus sequence characteristics of the *OXT* gene

Additional consensus sequences characterise the *OXT* promoter of domestic camelids, in particular, a TATA-box (-29/-24) and a CCAAT/enhancer binding protein-α (C/EBP-α) between the nucleotides -683/-674, both conserved between camelids and ruminants. Conversely, one nuclear factor 1 (NF1) binding site (-187/-178) is shared between camelids, cattle and buffalo, but it is not present in sheep or goat. NF1 consensus sequences characterise the promoters of many genes, where it may bind synergistically with some other transcription factors, including estrogen receptors [45].

Furthermore, one pituitary-specific transcription factor-1A (Pit-1A) consensus sequence (-833/-824) characterises the Old World camelids. The Pit-1 trans-activates the promoters of the cognate peptide hormone-encoding genes including the thyroid-stimulating hormone-β (TSH-β) [46], which indirectly promotes the oxytocin gene expression. Conversely, at least three binding sites (SP-1 at -905/-896, C/EBP-δ at -730/-721and AP-2 at -20/-11) are typical of the SACs.

Seven polymorphic sites were detected in the promoters of SACs (Table 3). The bioinformatics analysis showed that only the mutation MF464535:g.622C>G found in alpacas affects a putative regulatory binding site. In fact, the presence of guanine is responsible for the lack of a consensus sequence for the specificity protein 1 (SP1) transcription factor between the nucleotides -291/-281. This sequence is well conserved among domestic camelids, and its location only 115 bp upstream the CHRE suggest a key role in the *OXT* gene expression. It is known that any change in the consensus sequence results in a different strength of the transcription factor binding and, consequently, in the expression. For instance, an *in vitro* assay test in sheep *OXT* promoter recently demonstrated that the C allele for the SNP g.438T>C falling into a binding site for the transcription factor Oct-1 negatively affected the promoter activity of the *OXT* gene [13]. Therefore, the present result in alpacas opens the way to new investigations on expression analysis never studied in this species so far.

**Fig 2:**
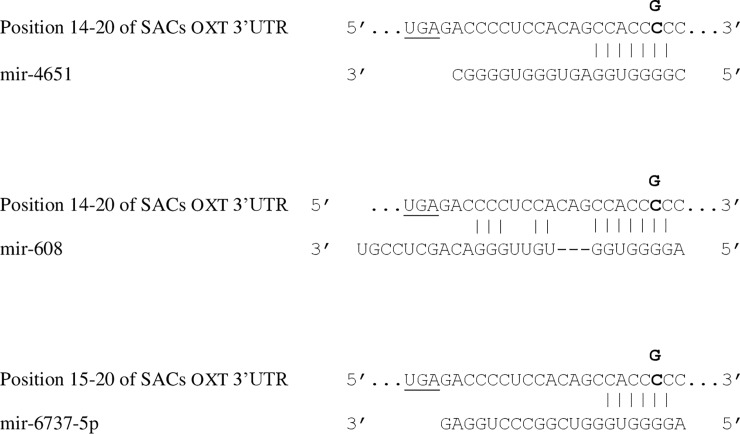
MicroRNA target sequences affected by SNP at 3’UTR. The transversion C>G (MF464535:g.1682C>G in alpacas and MF464534:g.1731C>G in llamas) falling 19 bp downstream the stop codon (underlined) affects different microRNA target sequences with 8mer (mir-4651, mir-608), 7mer-m8 (mir-6737-5p reported as example, but also mir-6819-5p) and 7mer-A1 (mir-6747-5p, mir-342-5p and mir-4664-5p). Binding of mature miRNAs are shown, whereas the site of the SNP is indicated in bold.

The 3’ end is also an important region for the control of the gene expression. In particular, microRNAs (miRNA) bind to the 3’ UTRs of the target genes and negatively regulate their expression [35]. Although the biological functions of most miRNA are unknown, it is estimated that more than 30% of protein-coding genes are regulated by miRNA [28]. The analysis of the 3’ flanking region in SACs showed genetic variability that could affect the gene regulation. A total of 6 polymorphic sites were found in the 3’end of the *OXT* gene, 2 in alpacas and 4 in llamas (Table 3). In particular, the mutations MF464535:g.1682C>G in alpaca and MF464534:g.1731C>G in llamas are conserved in SACs and they fall only 19 bp downstream the stop codon in the 3’UTR. Therefore, we have investigated whether such transversion could influence miRNA binding sites. Using the homologous human 3’UTR of the *OXT* gene as target, the bioinformatics analysis showed that the SNP in the 3’UTR of SACs *OXT* affects the binding sites of several miRNA (mir-4651, mir-608, mir-6737-5p, mir-6819-5p, mir-6747-5p, mir-342-5p and mir-4664-5p) characterised by different matching (8mer, 7mer-m8 and 7mer-A1) to the same canonical seed sequence CCACCCC (Fig 2).

The regulation of *OXT* gene expression by miR has already been reported in mouse hypothalamus by Choi et al. [47], who demonstrated that the miR-24 inhibits oxytocin production by targeting the boundary region between the coding sequence and 3′ UTR. Therefore, we cannot exclude the possibility that in camelids a region proximal to the stop codon might mediate *OXT* gene expression through the bound with the mir-4651 (http://www.targetscan.org/cgi-bin/targetscan/vert_71/targetscan.cgi?mirg=hsa-miR-4651), the mir-608 (http://www.targetscan.org/cgi-bin/targetscan/vert_71/targetscan.cgi?mirg=hsa-miR-608), or other novel candidate miRNAs. However, the biological significance of these putative candidates needs to be elucidated in future studies.

### Genotyping of the SNP g.622C>G in the alpaca promoter

**Fig 3:**
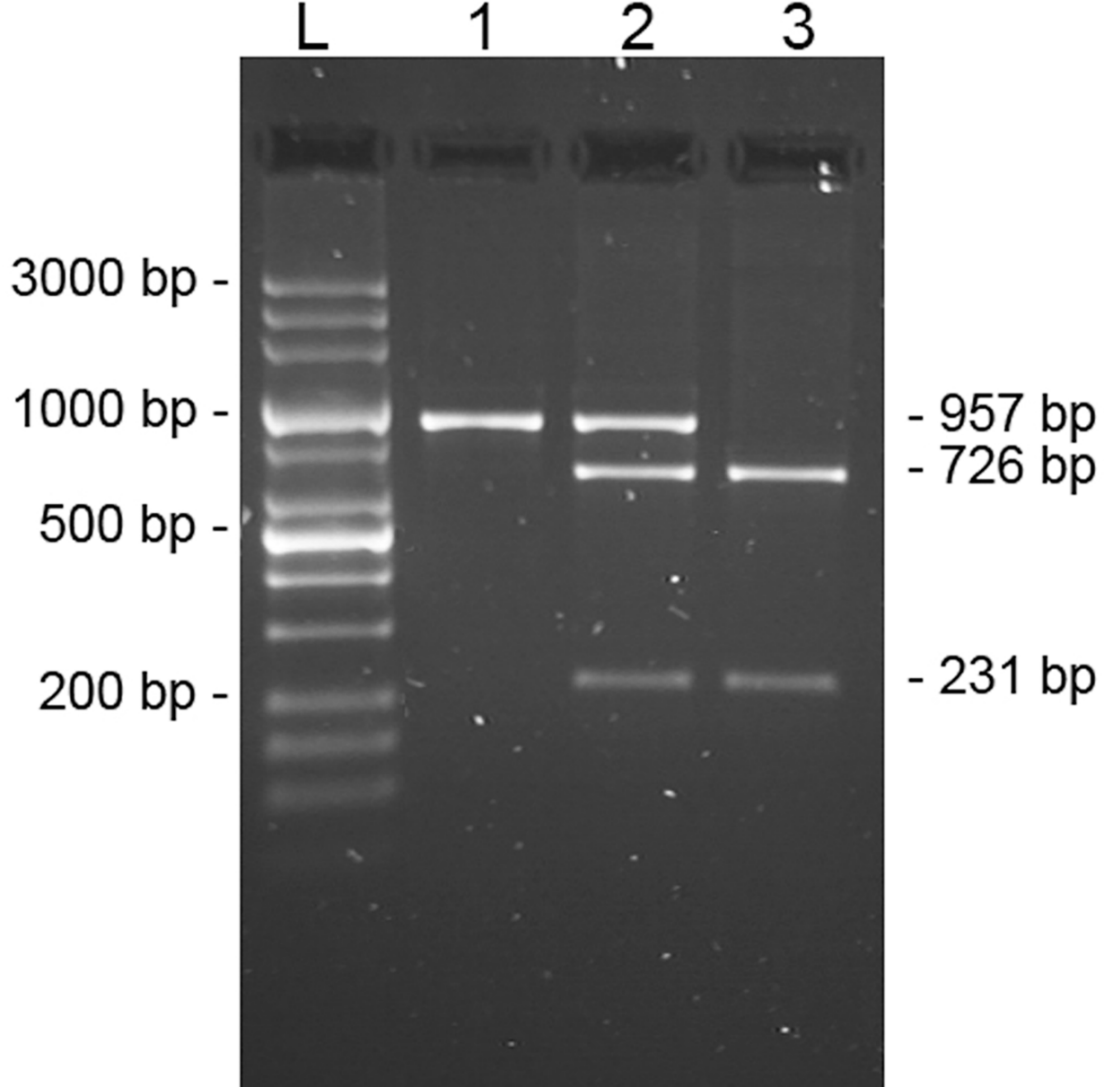
Genotyping of the g.622C>G at alpaca *OXT* promoter. Genotyping of the SNP MF464535:g.622C>G in the promoter region of *Vicugna pacos OXT* by *Bfo* I PCR-RFLP. Line 1, CC homozygous sample; line 3, GG homozygous sample; line 2, heterozygous sample. Line L, Mid Range DNA ladder 100bp-3kb (Jena Bioscience).

Since the SNP MF464535:g.622C>G in alpacas falls in the promoter region within a putative consensus site for the SP1 transcription factor, a PCR-RFLP protocol was set up for the quick genotyping of 71 alpaca samples. The digestion of the PCR product (957 bp) by *Bfo* I allows the identification of both alleles (Fig 3).

The homozygous CC is undigested, whereas the GG is restricted into two 2 fragments of 726 bp and 231 bp. The restriction pattern of the heterozygous genotype showed three fragments of 957 bp, 726 bp and 231 bp (Fig 3). The C allele had a frequency of 0.93 and the χ2 value (1.379) showed no evidence of departure from the Hardy-Weinberg equilibrium (P≤0.05). The low frequency of the G allele might be due to a genetic feature of the species or to a recent mutational event, but we can not exclude a selective disadvantage of this allele. In fact, the SNP g.622C>G falls also within a CpG island and the presence of the guanine (5’-CA**C**GGC-3’, herein underlined) creates a further putative methylation site for the upstream cytosine (in bold). DNA methylation is an epigenetic mechanism which regulates gene expression by influencing the recruitment and binding of regulatory proteins to DNA, and, in this respect, *OXT* does not represent an exception. In fact, data from the bisulphite sequencing of human genome (GRCh37/hg19 assembly) showed at least 14 methylated CpG sites within the first 350 bp upstream the exon 1 of the *OXT* gene (http://genome-euro.ucsc.edu/index.html), whereas a recent investigation to target and quantify DNA methylation across the promoter region of human *OXT* (Chr20, position 3052266–3053162) demonstrated an average methylation of 47% out of 9 CpG investigated sites [48]. In the same study, differences in the methylation level of the *OXT* promoter have been linked to several overt measures of human sociability.

These indications offer new opportunity of investigation to verify whether and how the genetic variant g.622G interferes in the gene transcription process and opens the way to new studies, including gene expression analysis, DNA methylation (bisulphite sequencing) and other epigenetic mechanism (post-translational modifications of histone proteins, noncoding RNAs, nucleosome positioning along the DNA). This will also increase the knowledge and annotation accuracy of the genome information also in domestic camelids.

### Conclusions

The present investigation has elucidated the structure of *OXT* gene in domestic camelids, providing fundamental knowledge on similarities and differences among them and with domestic ruminants. The polymorphisms described, provide useful information not only for *OXT* biodiversity itself, but also for possible future association studies with traits directly controlled by this hormone.

The identification in the promoter of the putative consensus sequences for the most important transcription factors, and in the 3’end of the presumed microRNA sequences, suggests the possible involvement of these motifs in the regulation of gene expression, thus opening future possibilities of investigation to verify the influence of the novel allelic variant in the *OXT* gene regulation.

## Supporting information

S1 Fig Homology between camelids and ruminants *OXT* gene. Homology between the complete nucleotide (nt) sequences of oxytocin-neurophysin I encoding (*OXT*) gene in domestic camelids (present work) with the corresponding OXT sequences of domestic ruminants. Numbering is relative to the first nucleotide of the first exon (+1) and dashes represent nt identical to those in upper line. The signal peptide is underlined, the coding region corresponding to the nonapeptide hormone is indicated in bold, whereas the neurophisin I is in bold italics. The tripeptide processing signal (GKR) is double underlined and asterisks indicate the stop codon. The deletion of an epta-nucleotide (GCTTTTG) and the duplication event of 21bp in *C*. *dromedarius* are indicated in bold-shade and wave-underlined respectively. The polyadenylation signal site is dot-underlined.
(DOC)

S2 Fig Homology between camelids and ruminants *OXT* promoter. Alignment and homology between the nucleotide sequences of the promoter region and partial exon 1 of domestic camelids (*C*. *dromedarius*, *C*. *bactrianus*, *V*. *pacos*, *L*. *glama*) *OXT* gene with the homologous 5’ flanking regions of the domestic ruminants (*B*. *taurus*, *B*. *bubalis*, *O*. *aries*, *C*. *hircus*). Numbering is relative to the first nucleotide of the first exon (+1). Dashes represent identical nucleotides to those in upper line. Putative consensus sequence for transcription factors are indicated in boxes. Shade boxes represent putative binding sites typical of the domestic ruminants (Cosenza et al., 2017). Genetic diversity within the camelids promoters is indicated in bold (Y = C/T; R = A/G; S = C/G, W = A/T).
(DOC)

## References

[1] Wu H, Guang X, Al-Fageeh MB, Cao J, Pan S, Zhou H, et al (2014) Camelid genomes reveal evolution and adaptation to desert environments. Nature communications 5: 5188 doi: 10.1038/ncomms6188 25333821 10.1038/ncomms6188

[2] FAO (2014) Food and Agriculture Organization of the United Nations. Statistics of live animals. URL http://www.fao.org/faostat/en/#data/QA.

[3] Volpato G, Howard P (2014) The material and cultural recovery of camels and camel husbandry among Sahrawi refugees of Western Sahara. Pastoralism 4: 7.

[4] Wardeh M (1993) The importance of the dromedary camel in the Arab countries al-IIbil.

[5] Kadwell M, Fernandez M, Stanley HF, Baldi R, Wheeler JC, Rosadio R, et al (2001) Genetic analysis reveals the wild ancestors of the llama and the alpaca. Proceedings of the Royal Society of London B: Biological Sciences 268: 2575–2584. 10.1098/rspb.2001.1774 PMC1088918 11749713

[6] Riek A, Gerken M (2006) Changes in llama (*Lama glama*) milk composition during lactation. Journal of dairy science 89: 3484–3493. doi: 10.3168/jds.S0022-0302(06)72387-6 16899683 10.3168/jds.S0022-0302(06)72387-6

[7] Wheeler JC (2012) South American camelids: past, present and future. Journal of Camelid Science 5: 1–24.

[8] Lollivier V, Guinard-Flament J, Ollivier-Bousquet M, Marnet P-G (2002) Oxytocin and milk removal: two important sources of variation in milk production and milk quality during and between milkings. Reproduction Nutrition Development 42: 173–186. 10.1051/rnd:2002016 12216962

[9] Bell CJ, Nicholson H, Mulder RT, Luty SE, Joyce PR (2006) Plasma oxytocin levels in depression and their correlation with the temperament dimension of reward dependence. Journal of Psychopharmacology 20: 656–660. doi: 10.1177/0269881106060512 16401658 10.1177/0269881106060512

[10] Striepens N, Kendrick KM, Maier W, Hurlemann R (2011) Prosocial effects of oxytocin and clinical evidence for its therapeutic potential. Frontiers in neuroendocrinology 32: 426–450. doi: 10.1016/j.yfrne.2011.07.001 21802441 10.1016/j.yfrne.2011.07.001

[11] Fuchs A-R, Fuchs F, Husslein P, Soloff MS, Fernstrom MJ (1982) Oxytocin receptors and human parturition: a dual role for oxytocin in the initiation of labor. Science 215: 1396–1398. 6278592 10.1126/science.6278592

[12] Gimpl G, Fahrenholz F (2001) The oxytocin receptor system: structure, function, and regulation. Physiological reviews 81: 629–683. doi: 10.1152/physrev.2001.81.2.629 11274341 10.1152/physrev.2001.81.2.629

[13] Cosenza G, Iannaccone M, Pico BA, Gallo D, Capparelli R, Pauciullo A (2017) Molecular characterisation, genetic variability and detection of a functional polymorphism influencing the promoter activity of *OXT* gene in goat and sheep. Journal of Dairy Research 84: 165–169. doi: 10.1017/S0022029917000097 28290268 10.1017/S0022029917000097

[14] Cosenza G, Pauciullo A, Mancusi A, Nicodemo D, Di Palo R, Zicarelli L, et al (2007) Mediterranean river buffalooxytocin-neurophysin I (*OXT*) gene: structure, promoter analysis and allele detection. Italian Journal of Animal Science 6: 303–306.

[15] Ivell R, Richter D (1984) The gene for the hypothalamic peptide hormone oxytocin is highly expressed in the bovine corpus luteum: biosynthesis, structure and sequence analysis. The EMBO journal 3: 2351 6209133 10.1002/j.1460-2075.1984.tb02139.x PMC557693

[16] Pauciullo A, Cosenza G, Steri R, Coletta A, Jemma L, Feligini M, et al (2012) An association between single nucleotide polymorphisms at the oxytocin locus and milk yield in Italian Mediterranean river buffalo. The Journal of Dairy Research 79: 150–156. doi: 10.1017/S0022029911000914 22280971 10.1017/S0022029911000914

[17] Shuiep E, Giambra IJ, El Zubeir IEYM, Erhardt G (2013) Biochemical and molecular characterization of polymorphisms of αs1-casein in Sudanese camel (*Camelus dromedarius*) milk. International Dairy Journal 28: 88–93.

[18] Kappeler S, Farah Z, Puhan Z (1998) Sequence analysis of *Camelus dromedarius* milk caseins. Journal of Dairy Research 65: 209–222. 9627840 10.1017/s0022029997002847

[19] Pauciullo A, Giambra I, Iannuzzi L, Erhardt G (2014) The β-casein in camels: molecular characterization of the *CSN2* gene, promoter analysis and genetic variability. Gene 547: 159–168. doi: 10.1016/j.gene.2014.06.055 24973699 10.1016/j.gene.2014.06.055

[20] Pauciullo A, Shuiep E, Cosenza G, Ramunno L, Erhardt G (2013) Molecular characterization and genetic variability at κ-casein gene (*CSN3*) in camels. Gene 513: 22–30. doi: 10.1016/j.gene.2012.10.083 23154061 10.1016/j.gene.2012.10.083

[21] Erhardt G, Shuiep ETS, Lisson M, Weimann C, Wang Z, El Zubeir IEYM, et al (2016) Alpha S1-casein polymorphisms in camel (*Camelus dromedarius*). Tropical animal health and production 48: 879–887. doi: 10.1007/s11250-016-0997-6 26922739 10.1007/s11250-016-0997-6

[22] Saadaoui B, Bianchi L, Henry C, Miranda G, Martin P, Cebo C (2014) Combining proteomic tools to characterize the protein fraction of llama (*Lama glama*) milk. Electrophoresis 35: 1406–1418. doi: 10.1002/elps.201300383 24519815 10.1002/elps.201300383

[23] Pauciullo A, Erhardt G (2015) Molecular characterization of the llamas (*Lama glama*) casein cluster genes transcripts (*CSN1S1*, *CSN2*, *CSN1S2*, *CSN3*) and regulatory regions. PloS one 10: e0124963 doi: 10.1371/journal.pone.0124963 25923814 10.1371/journal.pone.0124963 PMC4414411

[24] Pauciullo A, Gauly M, Cosenza G, Wagner H, Erhardt G (2017) *Lama glama* αS1-casein: Identification of new polymorphisms in the *CSN1S1* gene. Journal of dairy science 100: 1282–1289. doi: 10.3168/jds.2016-11918 27939542 10.3168/jds.2016-11918

[25] Fitak RR, Mohandesan E, Corander J, Burger PA (2016) The de novo genome assembly and annotation of a female domestic dromedary of North African origin. Molecular ecology resources 16: 314–324. doi: 10.1111/1755-0998.12443 26178449 10.1111/1755-0998.12443 PMC4973839

[26] Erhardt G., Gu M., Wagner H., Di Stasio L., Pauciullo A. *Vicugna pacos* αs1-casein: identification of new polymorphisms at the *CSN1S1* gene; 2017 6 12–14; Assisi, Italy. pp. 36. 10.3168/jds.2016-11918 27939542

[27] Sambrook J, Russell DW (2001) Molecular Cloning: A Laboratory Manual: Cold Spring Harbor Laboratory Press.

[28] Lewis BP, Burge CB, Bartel DP (2005) Conserved seed pairing, often flanked by adenosines, indicates that thousands of human genes are microRNA targets. cell 120: 15–20. doi: 10.1016/j.cell.2004.12.035 15652477 10.1016/j.cell.2004.12.035

[29] Wang Z, Ding G, Chen G, Sun Y, Sun Z, Zhang H, et al (2012) Genome sequences of wild and domestic bactrian camels. Nature Communications 3: 1202 doi: 10.1038/ncomms2192 23149746 10.1038/ncomms2192 PMC3514880

[30] Lindblad-Toh K, Garber M, Zuk O, Lin MF, Parker BJ, Washietl S, et al (2011) A high-resolution map of human evolutionary constraint using 29 mammals. Nature 478: 476 doi: 10.1038/nature10530 21993624 10.1038/nature10530 PMC3207357

[31] Ruppert S, Scherer G, Schütz G (1984) Recent gene conversion involving bovine vasopressin and oxytocin precursor genes suggested by nucleotide sequence. Nature 308: 554–557. 6709064 10.1038/308554a0

[32] Burger PA (2016) The history of Old World camelids in the light of molecular genetics. Tropical animal health and production 48: 905–913. doi: 10.1007/s11250-016-1032-7 27048619 10.1007/s11250-016-1032-7 PMC4884201

[33] Pauciullo A, Knorr C, Perucatti A, Iannuzzi A, Iannuzzi L, Erhardt G (2016) Characterization of a very rare case of living ewe-buck hybrid using classical and molecular cytogenetics. Scientific reports 6: 34781 doi: 10.1038/srep34781 27698378 10.1038/srep34781 PMC5048133

[34] Balmus G, Trifonov VA, Biltueva LS, O’Brien PC, Alkalaeva ES, Fu B, et al (2007) Cross-species chromosome painting among camel, cattle, pig and human: further insights into the putative Cetartiodactyla ancestral karyotype. Chromosome research 15: 499–514. doi: 10.1007/s10577-007-1154-x 17671843 10.1007/s10577-007-1154-x

[35] Cannell IG, Kong YW, Bushell M (2008) How do microRNAs regulate gene expression?: Portland Press Limited. 10.1042/BST0361224 19021530

[36] Safe S, Kim K (2004) Nuclear receptor-mediated transactivation through interaction with Sp proteins. Progress in nucleic acid research and molecular biology 77: 1–36. doi: 10.1016/S0079-6603(04)77001-4 15196889 10.1016/S0079-6603(04)77001-4

[37] Safe S (2001) Transcriptional activation of genes by 17β-estradiol through estrogen receptor-Sp1 interactions. Vitamins & Hormones 62: 231–252. 11345900 10.1016/s0083-6729(01)62006-5

[38] Richard S, Zingg H (1991) Identification of a retinoic acid response element in the human oxytocin promoter. Journal of biological chemistry 266: 21428–21433. 1657967

[39] Richard Sp, Zingg H (1990) The human oxytocin gene promoter is regulated by estrogens. Journal of biological chemistry 265: 6098–6103. 2108152

[40] Kimura T, Saji F, Nishimori K, Ogita K, Nakamura H, Koyama M, et al (2003) Molecular regulation of the oxytocin receptor in peripheral organs. Journal of molecular endocrinology 30: 109–115. 12683935 10.1677/jme.0.0300109

[41] Adan R, Cox JJ, Beischlag T, Burbach J (1993) A composite hormone response element mediates the transactivation of the rat oxytocin gene by different classes of nuclear hormone receptors. Molecular endocrinology 7: 47–57. doi: 10.1210/mend.7.1.8383287 8383287 10.1210/mend.7.1.8383287

[42] Adan R, Cox J, Van Kats J, Burbach J (1992) Thyroid hormone regulates the oxytocin gene. Journal of biological chemistry 267: 3771–3777. 1371278

[43] Ang H, Ivell R, Walther N, Nicholson H, Ungefroren H, Millar M, et al (1994) Over-expression of oxytocin in the testes of a transgenic mouse model. Journal of endocrinology 140: 53–62. 7511154 10.1677/joe.0.1400053

[44] Burbach J, da Silva SL, Cox JJ, Adan R, Cooney AJ, et al (1994) Repression of estrogen-dependent stimulation of the oxytocin gene by chicken ovalbumin upstream promoter transcription factor I. Journal of biological chemistry 269: 15046–15053. 8195142

[45] Pjanic M, Pjanic P, Schmid C, Ambrosini G, Gaussin A, Plasari G, et al (2011) Nuclear factor I revealed as family of promoter binding transcription activators. BMC genomics 12: 181 doi: 10.1186/1471-2164-12-181 21473784 10.1186/1471-2164-12-181 PMC3082249

[46] Andersen B, Rosenfeld MG (1994) Pit-1 determines cell types during development of the anterior pituitary gland. A model for transcriptional regulation of cell phenotypes in mammalian organogenesis. Journal of biological chemistry 269: 29335–29335. 7961905

[47] Choi JW, Kang SM, Lee Y, Hong SH, Sanek NA, Young WS, et al (2013) MicroRNA profiling in the mouse hypothalamus reveals oxytocin‐regulating microRNA. Journal of neurochemistry 126: 331–337. doi: 10.1111/jnc.12308 23682839 10.1111/jnc.12308 PMC3716862

[48] Haas BW, Filkowski MM, Cochran RN, Denison L, Ishak A, Nishitani S, et al (2016) Epigenetic modification of OXT and human sociability. Proceedings of the National Academy of Sciences 113: E3816–E3823. 10.1073/pnas.1602809113 PMC4941462 27325757

