# Supplementary material for: Genetic characterization of the oxytocin-neurophysin I gene (OXT) and its regulatory regions analysis in domestic Old and New World camelids
Source: PLoS One. 2018 Apr 2;13(4):e0195407. doi: 10.1371/journal.pone.0195407 (PMC5880406; doi:10.1371/journal.pone.0195407)
Supplement: S1 Fig — Homology between the complete nucleotide (nt) sequences of oxytocin-neurophysin I encoding (OXT) gene in domestic camelids (present work) with the corresponding OXT sequences of domestic ruminants. Numbering is relative to the first nucleotide of the first exon (+1) and dashes represent nt identical to those in upper line. The signal peptide is underlined, the coding region corresponding to the nonapeptide hormone is indicated in bold, whereas the neurophisin I is in bold italics. The tripeptide processing signal (GKR) is double underlined and asterisks indicate the stop codon. The deletion of an epta-nucleotide (GCTTTTG) and the duplication event of 21bp in C. dromedarius are indicated in bold-shade and wave-underlined respectively. The polyadenylation signal site is dot-underlined. (DOC) [file pone.0195407.s001.doc]

***Promoter*** +1 **5’UT** ***Exon 1*** ***Signal peptide***

cttaaaaggccagacccgagagacggccgcagtccccgg cccggagaccagcgcgtctgcaccATGGCAGGTTCCAGCC 49

--------------------------------------- ----------------------t-------A--------- 49

--------------------------------------- -------c-------------------------------- 49

--------------------------------------- -------c-------------------------------- 49

-a---------g-g--g-------c-tg------g----a---c-gac-------ac-ca---------C--CCT----- 50

-a---------g-g--g-------t-tg------g----a---c-gac-------ac-ca---------C--CCT----- 50

-a-----c---g-g--gc------c-tg------g----a---c-gac-------ac-ca---------C--CCT----- 50

-a-----c---g-g--gc------c-tg------g----a---c-gac-------ac-ca---------C--CCT----- 50

***Oxytocin*** ***G K R***

TCGCCTGCTGCCTGCTCGGCCTCCTGGCGTTGACCTCCGCC**TGCTACATTCAGAACTGCCCCCTGGGC**GGCAAACGC***GCG*** 129

-------------------------------------------------------------------------------- 129

-------------------------------------------------------------------------G------ 129

-------------------------------------------------------------------------G--T--- 129

-----------------------------C-------------------C-----------------------GA-G--C 130

-----------------------------C-------------------C-----------------------GA-G--C 130

-----------------------------C-------------------C------------T----------GA-G--C 130

-----------------------------C-------------------C------------T----------GA-G--C 130

***Neurophysin I***

***GTGCTGGACCTCGACGTGCGCACG***GTGAGAG CCCCGCCCTCGACCCGTGGCTCTCCGGGCTGCCCGGCCCGCTGCCACA 208

-----------------------------CTT--------------------G-----------TC------------T- 209

-----------------------------CG -----------T--------G--GG-------TA-----C-------- 208

-----------------------------CG -----------T------------G-------CA-------------- 208

-C-------------------CA------ -T--G-T--T-CC-- 174

-C-------------------CA------ -T--G-T--T-CC-- 174

-C-------------------CA------ -T--G-T----C--- 174

-C-------------------CA------ -T--G-T----C--- 174

GGGTCGCCCCCGCCGCCCCCTTTCCCGCGCTGACCGCGTACCGGCCCCACCTAGCCTGGGAATCGAGGGAGCGGAGGAGC 288

----------------------------------------------------G--------------------------- 289

-------------------------------------------------------------------------------- 288

----------------------------C--------------------------------------------------- 288

T--------T-----C---C---C-G-T ----- -A----T---TG 219

T--------T-----C---C---C-G-T ----- -A----T---TG-- 221

---------T-----C---C---C-G-T ----- **R**A----T---TG-- 221

---------T-----C---C---C-G-T ----- -A----T---TG-- 221

TTTTGACTGCCCTCCTTCG ACCGCTTTTGAGCCCAAAGAGAGAGCCAGGGAGACCCGCCACCTCCCGCGCTCCTCCGG 366

-------------------G--T-------G------------------------------------------------ 368

------------------- ---AA-----G------------ --G-------------T--------------A-- 364

------------------- ---AA------------------ -GG--A----------T--------------A-- 364

-T-C---CT----- ------G---G-GG-GC-A-C--CA------------ --------G-----------TT 292

------T-C---CT----- ------G---G-GG-GC-A-C--CA------------ --------G-----------TT 299

------T-C---CT----- ------G---G-GG-GC-A-C--CA-----A------ -------GG-----------TT 299

------T-C---CT----- ------G---G-GG-GC-A-C--CA-----A------ -------GG-----------TT 299

CCGCCCTCGCCCGCCCGGCTCAGC CCCCCGCCCCACAGGGTCTCCCTCCCCGGCC GCTCCCCTCCC GCCCCCGGC 441

------------------------ -----A------------------------- -G--------- --------- 443

------------------------ -----A------------------------- -G--------- --------- 439

------------------------ -----A------------------------- -G--------- --------- 439

--------C------------C--AT----G---T-T-G---------GA---A---C-GC--------CT-T------- 372

--------C------------C--AT----G---T-T-G---------GA---A--**Y**C-GC--------CT-T------- 379

--------C------------C--AT----G---T-T-G---------GA---A---C-GC--------CT-T------- 379

--------C------------C--AT----G---T-T-G---------GA---A---C-GC--------CT-T------- 379

***Neurophysin I***

TCATCCCTTCCCTCCCACCAG***TGTCTCCCCTGCGGCCCCGGGGGCAAAGGCCGCTGCTTCGGGCCCAGCATCTGCTGCGG*** 521

-------C------------------------------------------------------------------------ 523

-------CG-------G--------------------------------------------------------------- 519

-------CG-------G--------------------------------------------------------------- 519

---C---CG-------G------C-------------------------------------------------------- 452

---C---CG-------G------C-------------------------------------------------------- 459

---C---CG-------G------C-------------------------------------------------------C 459

---C---CG-------G------C-------------------------------------------------------C 459

***Exon 2***

***GGACGAGCTGGGCTGCTTCGTGGGCACGGCCGAGGCGCTGCGCTGCCAAGAGGAGAACTACCTGCCGTCGCCCTGCCAGT*** 601

-------------------------------------------------------------------------------- 603

-------------------------------------------------------------------------------- 599

-----------------------------------------------G-------------------------------- 599

---T--------------------------------------------G------------------------------- 532

---T--------------------------------------------G------------------------------- 539

------------------------------------------------G------------------------------- 539

------------------------------------------------G------------------------------- 539

***CCGGCCAGAAGCCCTGCGGGAGCGGGGGCCGCTGCGCCGCCGCCGGCATCTGCTGCAGCCCGG***GTGAGTCGGGCAGGG 679

------------------------------T----------------------------------------------- 681

------------------------------------------------------------------------------ 677

----------------------------------------------G-----------------------C------- 677

-------------G-----------------------------------------------C-------G----C---CC 612

-------------G-----------------------------------------------C-------G----C---CC 619

-------------G-----------------------------------------------C-------G----C---CC 619

-------------G-----------------------------------------------C-------G----C---CC 619

GTCGAGAC GGGACCGGGGCTCCAGGACCAGGCGGGCTGGGCCAGGGCGGCCCTGACT 736

-C------ --------------T---- ----------------------------- 737

-C------ ---G----------G-A-----------C---------T---------- 734

-C------ ---G----------G-A-----------C---------T---------- 734

G-CG-G---CGGGGCCGGGCCGGCGGGGACC---G------GCCC-----G-C-T-C-GC-----G**K**------G-----C 692

G-CG-G---C ---G------GC-------G-C-T-C-GC----**Y**G-------G-----C 678

G-CG-G---C ---G------GC--G----G-C-T-C-GC---- G-------G-----C 677

G-CG-G---C ---G------GC--G----G-C-T-C-GC---- G-------G-----C 677

***Exon 3 ******

CGGCGTCTCTCTGTGCAG***ACGGCTGCCACGAGGACCCCGCCTGCGACCCTGAGGCCGCCTTCTCCCAGCACTGA***gaccgg 816

------------------------------C------------------------------------------------- 817

------------------------------C---T--------------C------------------------------ 814

------------------------------C---T--------------C------------------------------ 814

T----------CCC-------------G--CC--G--------------C---------------------------- 770

T----------CCC-------------G--CC--G--------------C---------------------------- 756

T----------CCC-------------G--CC--G--------------C---------------------------- 755

T----------CCC-------------G--CC--G--------------C---------------------------- 755

***3’UT* PolyA-signal**

ccggcccccgataccgtcggagcgcagccctcactccctctgtaatcatccccaggaattatgacaatgaaataaagccg 896

------------------------------------------------------------------------------t- 897

--------t--c---a----------a---------------------------------------------------- 893

--------t--c---a-------a------------------------------------------------------- 893

----cac-gc--c---------------a-------------a- 814

----cac-gc--c---------------a-------------a- 800

----cac-gc--c-**s**-------------a-------------a- 799

----cac-gc--c-**s**-------------a-------------a- 799

gttttttccccctcc 911 bp *B. taurus* (GenBank ID: X00502)

--------------- 912 bp *B. bubalis* (GenBank ID: AM234539)

------t------- 907 bp *C. hircus* (GenBank ID: LT592266)

------t------- 907 bp *O. aries* (GenBank ID: LT592265)

a--- ------- 825 bp *C. dromedarius* (present work GenBank ID: MF464533)

a--- ------- 811 bp *C. bactrianus* (present work GenBank ID: MF464532)

a--- --g---- 810 bp *V. pacos* (present work GenBank ID: MF464535)

a--- --g---- 810 bp *L. glama* (present work GenBank ID: MF464534)
