# Supplementary material for: Genetic characterization of the oxytocin-neurophysin I gene (OXT) and its regulatory regions analysis in domestic Old and New World camelids
Source: PLoS One. 2018 Apr 2;13(4):e0195407. doi: 10.1371/journal.pone.0195407 (PMC5880406; doi:10.1371/journal.pone.0195407)
Supplement: S2 Fig — Alignment and homology between the nucleotide sequences of the promoter region and partial exon 1 of domestic camelids (C. dromedarius, C. bactrianus, V. pacos, L. glama) OXT gene with the homologous 5’ flanking regions of the domestic ruminants (B. taurus, B. bubalis, O. aries, C. hircus). Numbering is relative to the first nucleotide of the first exon (+1). Dashes represent identical nucleotides to those in upper line. Putative consensus sequence for transcription factors are indicated in boxes. Shade boxes represent putative binding sites typical of the domestic ruminants (Cosenza et al., 2017). Genetic diversity within the camelids promoters is indicated in bold (Y = C/T; R = A/G; S = C/G, W = A/T). (DOC) [file pone.0195407.s002.doc]

AGTCTGTCTTTCTTTTATTTTTCATTATTTTCGTTTGGAGATACCGTAC -917

---------G--------------------------------------- -917

----------------------C-------A---------------- -915

TGCTGAGCACCTCCCTGTTCATGGCAATAATGAC-AACAC-C-ACAG-GG-AGTC-CTC----T---AATT--TTTC-TT -914

-AGTC-CTC----T---AATT--TTTC -895

CCTCCCTGTTCATGGCAACACTGAC-AACAC-C-AC-G-GG-AGTC-CTC----T---AATT--TTTC -893

CCTCCCTGTTCATGGCAACACTGAC-AACAC-C-AC-G-GG-AGTC-CTC----T---AATT--TTTC -892

SP1

ATTAAAAATGGGGGGAAAA AAA CAAGAAATCATTGTTTTTAAATGCATAA AATGAAAGACATGGAATTA -848

------------------- --- ---------------------------- ------------------- -848

--------------GGG --CCC----------------**W**----------- ---------T--------- -844

---------------GGGG --CCC---------------------------- ---------T--------- -844

C--TTTTT-TTTAAATT-TTTTC-TTTT----T-CTG-ACA--AA-------A-- CA--CA---------A----- -838

-TTTT----T-CTG-ATA--AA-------A--CAATCA--CA---------A----- -838

-TTTT----T-CTG-ACA--AA-------A--AAATCA--CA---------A----- -836

-TTTT----T-CTG-ACA--AA-------A--AAATCA--CA---------A----- -835

C/EBP-α

Pit-1A

CAAAAGAAACCAGTTACATTTATGATCAAGCATCTAAATAGTAACACATTTGTGATACTGTCACAAATGTGCTCCATTAT -768

-------------------------------------------------------------------------------- -768

----------------T-----------------A--------------C-------------T---------------- -764

----------------T-----------------A--------------C-------------T---------------- -764

---------A---C--T-----------G-T---A-----T---A-------A-----A--------------A-T---- -758

-------------C--T-----------G-T---A-----T---A--G----A-----A--------------G-T---- -758

G-----G------C--T-----------G-T---A-----TC--A--G----A-----A--------------G-T---- -756

G-----G------C--T-----------G-T---A-----TC--A--G----A-----A--------------G-T---- -755

C/EBP-δ

TTAAAGCATCAGATAACAAGATCGAGTGGCAGG TCCAGTAACTGCCGTAATGTCAAAGTGATGATGAGTCTGATTT -692

--------------------------------- ------------------------------------------- -692

----------------------------- --- ----A-------------------------- --------- -692

----------------------------- --- ----A-------------------------- --------- -692

-- -------------T---------TCGG----A---T --A----T---C------A-----C-----G- -688

-- ------------TT---------TCCG----A---T --A----T---C------A-----C-----G- -688

-- --C----------T---------ACGG----A---T --A----T---C------AG----C-----G- -686

-- --C----------T---------ATGG----A---T --A----T---C------AG----C-----G- -685

C/EBP-α

TTAGAGACATTTGCAATAGCTGT AATGGGATAGGAGGAGATCTGTCATTTTCATTTGTGACAAAGCCATAGGGATTG -615

----------------------- ------------------------------------------------------ -615

----------------C------ -------------A---------------------------------------- -615

----------------C------ -------------A---------------------------------------- -615

-----------------------TGT---------------T---A-C-----CA-------------T--C---T---- -608

-----------------------TGT---------------T---A-C-----CA---------G---T--C---T---- -608

---------------G-------TGT----CA---------T---A-C-----CAG--------G---T--C---T---- -606

---------------G-------TGT----CAG--------T---A-C-----CAG--------G---T--C---T---- -605

AP1 AP1

CAGATTCCACCGTGGTTTCTTGCCTACCTCACA GTTGAAGAAAAGGCTGAATTTCAGTTAGAAGTGAGAGAAAATAAAA -536

--------------------------------- ---------------------------------------------- -536

-------------------------C-**Y**----- ---A---G-------------------------------------- -536

-------------------------C------- ---A---G-------------------------------------- -536

TGA-------T-------G-------T--T-T-C-C-----G--------G-------------A--------------- -528

-GA-------T-------G----------T-T- -C-----G-------TG-------------A--------------- -529

--A-------T-------G----------T-T- -------G--------G------C------ -------------- -529

--A-------T-------G----------T-T- -------G---------------C------ -------------- -528

SP1 SP1

TCTTTTCCTATCTGAGTTCCAGGTGCAAGGAAGTGGAAGCTGGTGTTGGGAGTGGGGGCAGAGTTCCAGGAAATTTGGAT -456

-------------------------------------T------------------------------------------ -456

------------C------------------------T----T------------------G----------------G- -456

------------C------------------------T----T------------------G----------------G- -456

-------A----CA---G--T-C ----T-T------G-----CC---CTG-G------------CA-AT- -458

-------A----CA---G--T-C ----T-T------G-----CC---CTG-G------------CA-AT- -459

-----Y-A----CA--AG--TCC-C--G-T--A----T-T------A-----CC---CTG-G------------CA-AT- -449

-----Y-A----CA--AG--TCC-C--G-TT-A----T-T------A-----C----CTG-G------------CA-AT- -448

Oct-1

TCAGGAGGAGGAGAGCTTCCTTGCAGAGGGGAACTGGGATGCGCCTGTGGCTGGCAAAGGGGGCAGATGGGG -384

------------------------------------------------------------------------ -384

-------**R**---------------------A---------C---------------C---------------- -384

-----------------------------A---------C---------------C---------------- -384

------A---A- -C-G-----T-------G--A--GG-T-TA-----T----C---A------------AAGTTCTG -381

------A---A- ---G-------------G--A--GA-T-TA-----T----C---A------------AAGTTCTG -382

------A---A- ---G-------------G--A--GG-T-TG-----T----C---A------------GAGTCCTA -372

------A---A- ---G------------ G--A--GG-T-TG-----T----C---A----------A-GAGTCCTG -371

SP1 SP1 SP1

AGTCCCTTCTGGCCTGGAGCGGCCCAAGTGTCCTCCCTGGCACCCCCTCCAAAGGTATTGGGAGTGGGCTACATAG -308

----------------C----------------------------------------------------------- -308

---------------------------------------A-C--T--C-------------------A-------- -308

-------------------------**R**-------------A-C--T--C-------------------A-------- -308

AACG-A--T-----T-------- -----G-----T-AT----A-C--T-AC--C--C-G----A---------C----- -302

AACG-A--T-----CT------- -----G-----T-AT----A-C--T-AC--C----G----A---------C----- -303

AACC-A--T-----C-------- -----G-----T-AT----A-C--T-AC--C--A-G-A--A--- -- -303

AACC-A--T-----C-------- -----G-----T-AT----A-C--T-AC--C--A-G-A--A--- -- -302

AP-1

SP1

TTCTTGGTTGGGCCACCGCGCCCCCCATACTCCCTATTCCACCCTAGGACCCAGGCCTCCGCACCG CTCCCATCCTCGA -229

------------------------------------------------------------------ -------A----- -229

----------------**S**---------G------------------------------A----**R**T-- ------------- -229

--------------------------G------------------------------A----**R**T-- ------------- -229

-C--C---------G-A-- -------------G-C-------------T-----A------A-G-----TG--CA-- -225

-C--C---------G-A-- -------------G-C-------------T-----A----TTA-G-----TG--CA-- -226

-C--C---------G-A-- -------------G-C-----G-------------A------A-G-----TG--CA-- -226

-C--C---------G-A-- -------------G-C-------------------A------A-G-----TG--CA-- -225

NF1 CHRE

GCGGCTTCCTTCGCCCA GCGGCACTCGCTATCATCACCGGT CCATTAGCCATTAGCCTGATGCGGTGACCTTGACCCG -151

----------------- ------------------------ ------------------------------------- -151

--A-------------- ------------------------ ----------------C-------------------- -151

--A-------------- ------------------------ ----------------C-------------------- -151

--T----G----C---- --A---------------T--C-GA---------------A -ACA-A----------- -150

--T----G----C----A--A---------------T--C-GA---------------- -ACA-A----------- -150

--T----G----C--T- -----G------------T--C--AA--------------A -ACA-A----------- -151

--T----G----C--T- ------------------T--C--AA--------------A -ACA-A----------- -150

SP1 ERE ERE

GGCCTGGCCGCTGCAAATGAGTGGGTGCGCCGGGGGCGCAGGGCTTGACCCGCAGCGGCCCGCTGTGACCAGCCATGCGG -71

-------------T------------------------------------------------------------------ -71

---G---------------------C------------------------------------------------------ -71

---G---------------------C------------------------------------------------------ -71

- -ACA---C-----------G---C------------------C--------------G------------T------- -71

- -ACA---T-----------G---C------------------C--------------G------------T------- -71

- -ACA---C-----------G---C------------------C--------G-----G------------T------- -72

- -ACA---C-----------G---C------------------C--------------G---A--------T------- -71

SP1 TATA-box AP2 +1

CGGCCCTCTTA GACACGGCTCCGCCGGCGCGGCCCCCGGGCATAAAAGGCCGGGCCGGAGAGACCGTGGCagtcgccgg 9

----------- -----------------------------------------------------T-------------- 9

----------- ------------------------------------C---------C--------------------- 9

----------- ------------------------------------C---------C--------------------- 9

-T--------- ----TC-T------CA-------G------T---------A-A--C-------G-CC------c---- 9

-T--------- -----C-T------CA-------G------T---------A-A--C-------G-CC------c---- 9

-T---------A-----C-T------CA-------G------T---------A-A--C-------G-CC------c---- 9

-T--------- -----C-T------CA-------G------T---------A-A--C-------G-CC------c---- 9

5’UTR Exon 1 *M A G L S*

accccggacccagcgcacccacacc**ATGGCCGGCCTCAGC** 49 *C. dromedarius* (MF464533)

---------------------------------------- 49 *C. bactrianus* (MF464532)

---------------------------------------- 49 *V. pacos* (MF464535)

---------------------------------------- 49 *L. glama* (MF464534)

---g-aga-------gt-tg--t------**AA-TTC**---- 48 *B. bubalis* (AM234538; AM234539)

---g-aga-------gt-tg---------**A--TTC**---- 48 *B. taurus*  (AB481096)

---g-ag--------gt-tg---------**A--TTC**---- 48 *O. aries* (LT592265)

---g-ag--------gt-tg---------**A--TTC**---- 48 *C. hircus* (LT592266)

*M A G S S*
